# Impact of storage environment on the efficacy of hermetic storage bags

**DOI:** 10.1016/j.jspr.2017.03.008

**Published:** 2017-05

**Authors:** Brett Lane, Charles Woloshuk

**Affiliations:** Department of Botany and Plant Pathology, Purdue University, 47907 West Lafayette, IN, USA

**Keywords:** PICS bags, Aflatoxin, Maize storage

## Abstract

Small hermetic bags (50 and 100 kg capacities) used by smallholder farmers in several African countries have proven to be a low-cost solution for preventing storage losses due to insects. The complexity of postharvest practices and the need for ideal drying conditions, especially in the Sub-Sahara, has led to questions about the efficacy of the hermetic bags for controlling spoilage by fungi and the potential for mycotoxin accumulation. This study compared the effects of environmental temperature and relative humidity at two locations (Indiana and Arkansas) on dry maize (14% moisture content) in woven polypropylene bags and Purdue Improved Crop Storage (PICS) hermetic bags. Temperature and relative humidity data loggers placed in the middle of each bag provided profiles of environmental influences on stored grain at the two locations. The results indicated that the PICS bags prevented moisture penetration over the three-month storage period. In contrast, maize in the woven bags increased in moisture content. For both bag types, no evidence was obtained indicating the spread of *Aspergillus flavus* from colonized maize to adjacent non-colonized maize. However, other storage fungi did increase during storage. The number of infected kernels did not increase in the PICS bags, but the numbers in the woven bags increased significantly. The warmer environment in Arkansas resulted in significantly higher insect populations in the woven bags than in Indiana. Insects in the PICS bags remained low at both locations. This study demonstrates that the PICS hermetic bags are effective at blocking the effects of external humidity fluctuations as well as the spread of fungi to non-infected kernels.

## Highlights

• The environmental effects on hermetically stored maize are examined.
• PICS bags block the external moisture from grain during storage.
• Fungal growth and kernel infection are greatest during storage in woven bags.
• Warmer environments result in more rapid loss in grain quality.

## 1 Introduction

Maize is one of the most important crops grown in Africa and the primary cereal grain (Smale et al., 2013). Post-harvest losses are a significant concern, reaching as high as 36% (Tefera, 2012). Insects are the major cause of dry matter loss; however, improper grain drying often results in damage by storage fungi and the risk of mycotoxin accumulation (Hell et al., 2000). The majority of smallholder farmers use traditional methods to handle and store their grain after harvest. Solar drying is effective, but it is often difficult to attain the targeted grain moisture when weather conditions are not favorable (Prakash and Kumar, 2013). Storage of the grain in woven bags is inexpensive but requires the application of insecticide (De Groote et al., 2013, Kamanula et al., 2010, Maina et al., 2016). Farmers have become more aware of the potential health issues associated with these insecticides, especially when the grain is stored within the home.

Over the past decade, the application of hermetic storage bags has been promoted in the Sub-Sahara region for the storage of maize and other vulnerable crops (Baoua et al., 2014, De Groote et al., 2013). The bags are made of plastic with low permeability to atmospheric gases. Respiration by the grain, insects, and fungi lead to a reduction in oxygen and an increase in carbon dioxide within the hermetic bag (Murdock et al., 2012). Within a short period of time, conditions become inhibitory to insect and fungal growth and development. Although these bags cost significantly more than the traditional woven bags, the need for insecticide applications is eliminated.

One of the concerns associated with storing maize in hermetic bags has been the efficacy for controlling the growth of mycotoxigenic *Aspergillus* species and the potential for aflatoxin accumulation during storage. Aflatoxins are potent carcinogens produced primarily by *A. flavus and A. parasiticus* (Woloshuk and Shim, 2013). Acute aflatoxicosis poses a serious risk of death, while chronic exposure is tied to cancer and a compromised immune system (Williams et al., 2004). High levels of aflatoxin contamination in Kenya maize in 2004 led to 125 deaths (Lewis et al., 2005). Because of these health risks, acceptable levels of aflatoxins in food and feed are regulated by most governments; however, significant levels are still found in many rural markets (Lewis et al., 2005).

The use of hermetic storage for the protection of grain from aflatoxin accumulation has been tested multiple times, often with conflicting results. Williams et al. (2014) demonstrated the use of PICS bags for the storage of maize at 27 °C in laboratory conditions, showing that PICS bags mitigate the growth of *A. flavus* and the accumulation of aflatoxin during storage as well as maintaining the initial moisture content of the maize. Similar results were reported for the storage of shelled peanuts in hermetic bags at 30 °C (Navarro et al., 2012). However, Fusseini et al. (2016) found an increase in maize moisture content as well as aflatoxin levels during storage in triple-layer hermetic bags across multiple temperatures under laboratory conditions. Their results also indicated that cooler temperature (16 °C) resulted in the largest increase in aflatoxin accumulation. This conflicting evidence furthers the debate on the efficacy of hermetic storage for the mitigation of accumulation of aflatoxin. Under field conditions in Brazil and Kenya, the number of *Aspergillus* spp. increased during storage, even in hermetic storage systems (Di Domenico et al., 2016, Maina et al., 2016, Viebrantz et al., 2016). However, aflatoxin accumulation during these storage experiments did not provide conclusive evidence about the efficacy of the hermetic systems.

In the study presented here, we determined the ability of *A. flavus* to grow, spread, and accumulate aflatoxin in storage bags by placing non-contaminated grain in small satchels adjacent to *A. flavus*-contaminated grain contaminated with aflatoxin. We compared the Purdue Improved Crop Storage (PICS) hermetics bags with polypropylene woven bags. PICS bags are a triple layer hermetic storage bag that are effective at protecting stored grains from damaging insect infestation (Baoua et al., 2014, Murdock et al., 2012). Our results demonstrate the efficacy of PICS bags for protecting the grain from moisture and temperature fluctuations during storage.

## 2 Materials and methods

### 2.1 Fungi and culture material

*Aspergillus flavus* strain NRRL-3557 was used for this study. The fungus was grown on potato dextrose agar (PDA) medium in culture plates (100 mm × 15  mm) at 30° C for five days. To produce *A. flavus*-colonized maize, kernels were soaked in water overnight, then placed in mushroom-spawning bags (www.gourmetmushroomsupply.com) and autoclaved twice (Tubbs et al., 2016). A culture of *A. flavus* grown for five days was finely chopped and added to 500 g of autoclaved maize. The inoculated maize was incubated for 2 days at 23 °C, after which the maize was washed with water to remove conidia and dried to a moisture content of 13%. Inoculated maize (100 g) was placed in small satchels made of mesh (5.5 cm diameter). For controls, non-inoculated maize was placed into satchels.

### 2.2 Storage bag preparation

Maize was grown within two miles of Buck Creek Elevator (Buck Creek, IN USA), where it was purchased for this experiment. The producer harvested the grain in the fall of 2014 and dried it before placing it in on-farm storage through the winter. The grain was graded as U.S. No. 5 Yellow Corn by Titus Grain Inspection, Inc. (West Lafayette, IN, USA), a USDA licensed grain inspector. Broken corn plus foreign material (BCFM) was determined to be 5.3%. At the time of purchase, the grain moisture content (MC) was 14.0% ± 0.06% dry weight based as determined by standard protocols (Standard, 2012).

The two types of storage bags used in this study were the triple-layer 50 kg PICS bags and single-layer 50 kg woven polypropylene bags. Bags were partially filled with 40 kg of maize, and three satchels of *A. flavus*-inoculated grain and three satchels of non-inoculated grain were placed in the middle of each bag. The satchels were separated by about 2.5 cm. Each bag also contained a humidity and temperature data logger (Lascar EL-USB-2, MicroDAQ, Contoocook, NH USA), which acquired data at 15-min intervals. Filled bags were placed in 31-gallon (117 L) galvanized, steel cans (68.6 cm H × 52 cm D) (Behrens Manufacturing, Winona, MN USA), which were modified by cutting four ventilation panels (41.9 cm H × 11.4 cm W) equidistant around the sides of the can. The panels were covered with screen (0.64 cm, 23-gauge steel) for rodent protection. Three replicates of the storage containers were placed at two locations and observed from June through September of 2015: the Agronomy Center for Research and Education (Purdue University, West Lafayette, IN, USA) and the Lon Mann Cotton Research Station (Marianna, AR, USA). At each location, two additional data loggers were placed outside of the bags but within the storage cans to record external humidity and temperature.

### 2.3 Sample and data collection

Maize samples were collected at the start of the experiment (zero time) and after 3 months of storage. After the storage period, grain in each bag was retrieved by hand and sieved through a 3/16 inch (4.76 mm) round-hole sieve. The material passing through the sieve was stored at −20° C until insects were analyzed. At the same time, grain samples (750 g) were collected from the top, middle and bottom regions of each bag. Moisture content of the maize was determined in triplicate for the top, middle, and bottom of each bag by dry weight methods according to ASABE standards (Standard, 2012).

To quantify surface fungi, five samples (30 g) were collected from each bag layer. Each sample was placed in a flask with 50 mL of 0.05% Triton X-100 solution and shaken for 1 min. The kernel wash was serially diluted and plated onto malt salt agar (MSA) medium (McDonough et al., 2011), and incubated for 3 days at 30 °C to obtain fungal colony counts (CFU). The resulting washed kernels were transferred to a beaker containing bleach (5% NaOCl), stirred for 1 min, and washed twice with sterile water. For each layer in the bag, three samples of 50 kernels were plated onto MSA medium. Kernel infection rate was determined by counting the number of kernels exhibiting fungal growth after three days of incubation at 30 °C. The maize in the three non-inoculated grain satchels in each bag were combined and mixed well. A subsample of 50 g was taken for aflatoxin analysis, and the remaining grain was surface sterilized and plated onto MSA medium to obtain a kernel infection rate.

Maize collected from the top, middle, and bottom of each bag was also tested for germination as described by (Williams et al., 2014) except the incubation temperature was 23 °C. For each layer, data were collected from three replicates of 50 kernels.

### 2.4 Aflatoxin analysis

For aflatoxin analysis, a 50 g sample of maize was ground in a coffee grinder, and three subsamples (0.5 g) were extracted overnight in 2 mL of acetonitrile. The extracts were filtered (4 mm × 0.45  μm Iso-Disc Filter, Supelco, Bellefonte, PA) before injection into a Shimadzu HPLC (Shimatzu Scientific Instruments, Inc. Kyoto, Japan) equipped with an analytical C18 column (5 μm, 4.6 mm × 150 mm, Alltech Econosphere), a post-column PHRED (Aura Industries, New York, NY), and a Shimadzu fluorometer (360 nm excitation and 440 nm emission). The mobile phase consisted of water, acetonitrile, and methanol (68:24:8, v:v:v). Aflatoxin B1 was quantified by comparing peak areas with a standard (Sigma Chemical Co., St. Louis, MO) over a range of 1–100 ng aflatoxin B1/ml.

### 2.5 Statistical analyses

SAS 9.4 (SAS Institute Inc., Cary, NC) with PROC GLM and CONTRAST was used to analyze data and determine statistical significance.

## 3 Results

### 3.1 Temperature, relative humidity, and grain moisture

Environmental conditions at each storage location were recorded by two data loggers placed outside the storage bags. The day/night temperatures in Arkansas were considerably warmer than in Indiana (Table 1). A high daytime temperature of 34 °C was measured in Arkansas in all three months of the study and a total of 66 days experienced temperatures above 30 °C. In contrast, the highest temperature recorded at the Indiana site was 30 °C, which was experienced on one day in July. The nighttime temperatures were cooler in Indiana than Arkansas, where 51 nighttime temperatures were below 20 °C. In July, Arkansas nights were very warm, with none below 20 °C and five nights with lows above 27 °C.

**Table 1:** Summary of temperatures recorded at experimental sites in Arkansas and Indiana in the summer of 2015.

Month	Highest/Lowest		Days with High ≥ 30 C		Nights with Low ≥ 27 C		Nights with Low < 20C	
	AR	IN	AR	IN	AR	IN	AR	IN
July	34/22	30/15	26	1	5	0	0	17
August	34/21	28/14	20	0	0	0	5	20
September	34/14	29/11	15	0	0	0	8	14

**Fig. 1:**
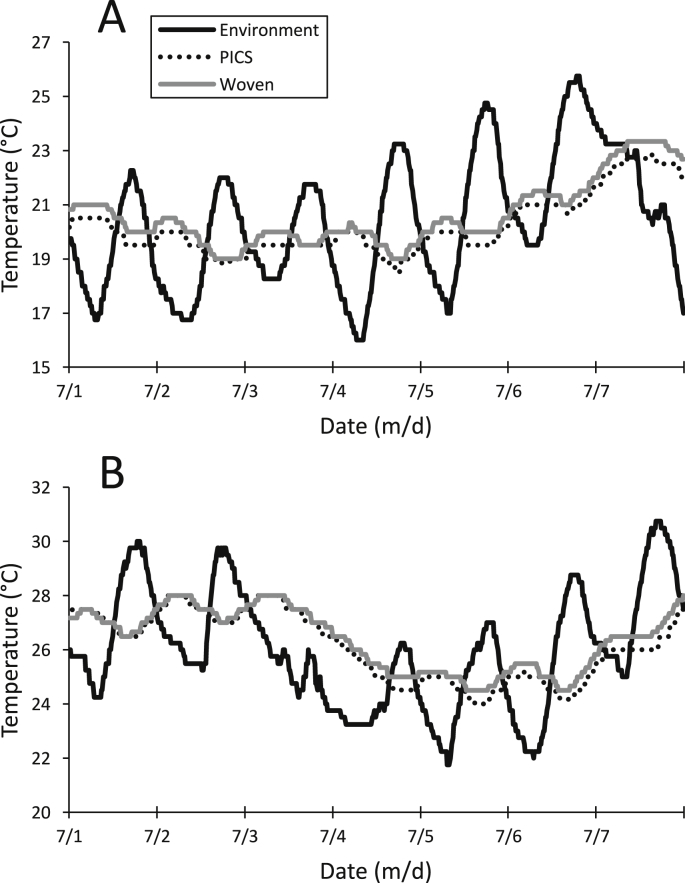
Oscillation of the daily temperature outside (environment) and inside the storage bags at A) Indiana and B) Arkansas. Data represent the average values from two data loggers outside and three inside the replicate bag treatments.

**Fig. 2:**
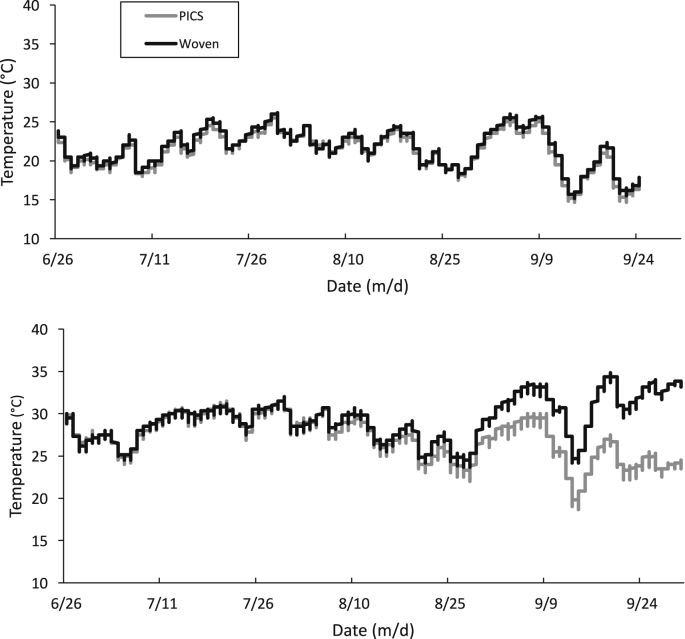
Temperature inside the bags stored in A.) Indiana and B.) Arkansas. Data represent the average values from data loggers inside the replicate bag treatments.

The effect of the outside day/night temperatures on the grain mass was monitored with data loggers within each storage bag. Warming and cooling of the grain mass followed an oscillation similar to the outside day/night temperatures with a slight delay for heat transference (Fig. 1A and B). Over the three-month study, little difference was observed between the PICS and woven bags stored in Indiana (Fig. 2A and B). In contrast, grain in the woven bags in Arkansas was warmer in late August than in the PICS bags, likely due to the increase in insect populations (Fig. 2B). Despite this increase, the day/night oscillation was discernible.

**Fig. 3:**
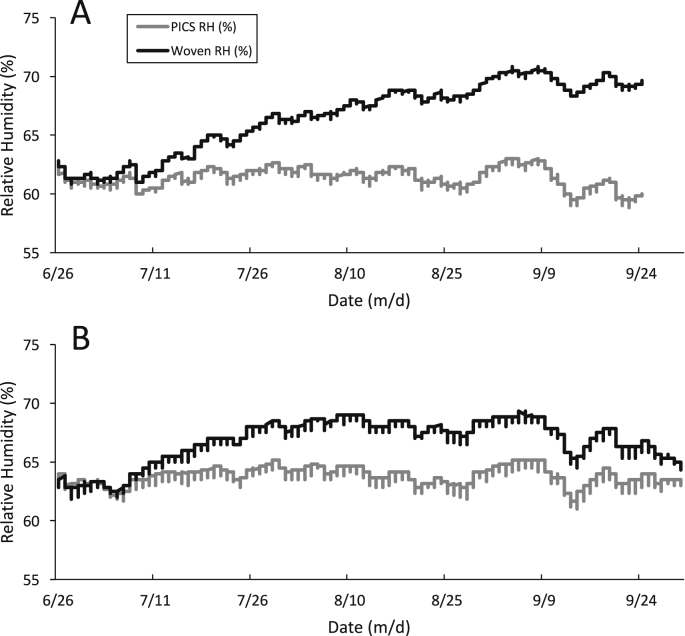
Relative humidity inside the bags stored in A.) Indiana and B.) Arkansas. Data represent the average values from data loggers inside the replicate bag treatments.

During the storage period, the average relative humidity (RH) was higher in Indiana than in Arkansas (Table 2). Arkansas had 27 days, including 20 days in September, when the average RH was less than 65%. In contrast, Indiana experienced only 2 days when the average RH was less than 65%. Indiana also experienced 13 days with the average RH over 85% compared to Arkansas with only 1 day. Fluctuations in the daily RH of the outside environment had little immediate effect on the data loggers within the storage bags. However, the RH steadily increased within the woven bags stored in Indiana (Fig. 3A). In Arkansas, the RH in the woven bags increased during July, leveled off in August, and decreased during September (Fig. 3B). In contrast, the RH in the PICS bags at both locations remained unaffected by the environmental RH, remaining below 65% (Fig. 3A and B). The increase in humidity within the woven bags correlated with rewetting of the stored grain. After three months, grain stored in the woven bags increased from the initial 14.03% (SE = ± 0.4) to 15.91% (SE = ± 0.3) in Indiana and to 14.94% (SE = ± 0.9) in Arkansas. In the PICS bags, the moisture content of the grain after storage was 14.24% (SE = ± 0.2) in Indiana and 14.30% (SE = ± 0.2) in Arkansas. Grain moisture content values from PICS bags between the storage locations were not significantly different. For the woven bags, the grain moisture was significantly higher (P < 0.01) in Arkansas than in Indiana. Also, the grain moisture in PICS bags was uniform between the top, middle, and bottom layers at both locations. The moisture content was also uniform in the woven bags in Indiana, but moisture was significantly higher (P < 0.01) at 15.4% in the bottom layer of the woven bags in Arkansas. Further analysis showed no significant difference (P < 0.01) between the replicates of each treatment.

**Table 2:** Summary of relative humidity recorded at experimental sites in Arkansas and Indiana for the summer of 2015.^a^

			Number of days							
	Highest/Lowest		>85%		84%–75%		74%–65%		<65%	
	AR	IN	AR	IN	AR	IN	AR	IN	AR	IN
July	91%/48%	100%/52%	0	10	14	16	16	5	1	0
August	93%/43%	95%/51%	1	3	9	11	15	17	6	0
September	87%/27%	92%/47%	0	0	3	9	7	13	20	2

a Average daily relative humidity was calculated from 96 data points collected over 24 h period.

### 3.2 Insects

The initial grading of the maize used in this study did not reveal any evidence of insect infestation. However, after three months of storage, insects were present under all treatments (Table 3). When the woven bags in Arkansas were opened, a large number of adult Indian meal moths (*Plodia interpunctella*) were present at the grain surface. Only a few moths were observed in the woven bags stored in Indiana. No Indian meal moths were observed when the PICS bags were opened. All grain in the storage bags was passed through a sieve to collect the insects. In the three woven bags from the Arkansas location, 841 (SE = ± 62) insects per kg of maize were counted, which was significantly (P < 0.01) more than the 10 (SE = ± 1.2) insects per kg of maize in the PICS bags. Maize weevil (*Sitophilus zeamais*) was the predominate insect. Also present were parasitic wasps, rusty grain beetles (*Cryptolestes ferrugineus*), and unidentified beetles and insect larvae. Insect populations were lower in Indiana, with 25 (SE = ± 1.1) and 5 (SE = ± 0.8) insects per kg of maize in the woven and PICS bags, respectively. There was a significant difference (P < 0.01) between the bag types, and the insects identified were similar to those found in Arkansas.

**Table 3:** Number of insects in PICS and woven bags after three months of storage in Indiana and Arkansas.^a^

	Indiana		Arkansas	
	PICS	Woven	PICS	Woven
Total	5.0 ± 0.8 A^c^	25 ± 1.1 A	10 ± 1.2 A	841 ± 61.7 B
Weevils	3.9 ± 0.8 A	19 ± 2.1 A	2.1 ± 0.5 A	589 ± 37.4 B
Beetles	0.1 ± 0.06 A	3.5 ± 1.0 A	7.0 ± 1.3 A	66 ± 5.4 B
Parasitic Wasps	0 A	0 A	0 A	132 ± 14.6 B
IMM^b^	0 A	0.1 ± 0.05 A	0 A	4.2 ± 0.6 B
Larvae	1.0 ± 0.3 A	1.4 ± 0.6 A	0.7 ± 0.2 A	46 ± 10.4 B

a Values are the mean number of insects per kg of maize ± SE.

b Adult Indian Meal Moths.

c Letters represent significance (P < 0.01) across each row.

### 3.3 Surface fungi and infected kernels

Grain collected from the top, middle, and bottom layers of each storage bag was analyzed to determine the number fungi on the kernel surface and the number of infected kernels. The number of fungi washed from the surface of the initial grain used for storage was 1.3 × 10^3^ (SE = ± 302) CFU/g of maize. *Fusarium* and *Penicillium* were the major genera identified on the culture medium. After three months of storage, the most predominant fungi were *Fusarium*, *Penicillium*, and *Aspergillus* species. *Alternaria* species were also observed as well as other sporulating fungal colonies and yeast-like fungal colonies. In Indiana, 9.8 × 10^3^ (SE = ± 637) CFU/g of maize was washed from the grain stored in the woven bags, compared to 3.7 × 10^3^ (SE = ± 878) CFU/g of maize in the PICS bags. The number of fungi on the maize in the Arkansas PICS bags was not significantly different (P < 0.01) than the Indiana PICS bags (2.8 × 10^3^ (SE = ± 481) CFU/g of maize). In contrast, grain in the woven bags in Arkansas had 3.0 × 10^5^ (SE = 1.0 × 10^5^) CFU/g of maize. Our analysis indicated no significant difference in the number of surface fungi between layers except in the woven bags stored in Arkansas (P < 0.01). In two of the replicate bags, the bottom layer had significantly (10-fold) more surface fungi than other layers, and in the third replicate bag, the middle had significantly more fungi than other layers. Overall there was no significant difference (P < 0.01) between replicates of each bag type for a given environment.

**Fig. 4:**
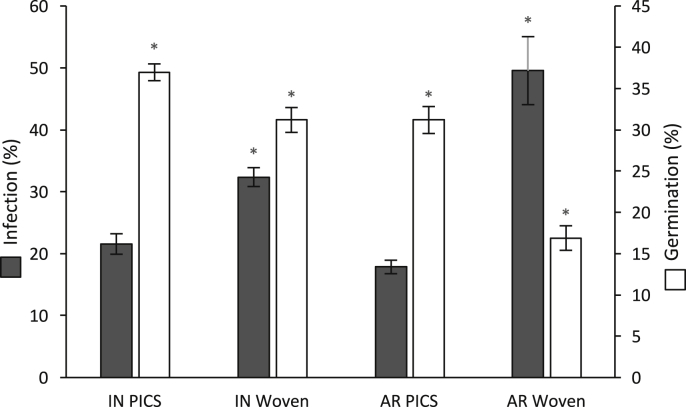
Effect of bag type on kernel infection and seed germination after three months of storage. Data are the mean value of three replicate bags, and bars represent SE. Symbols (*) represent significant differences (P < 0.01) from the initial infection (22% ± 2.8%) and germination (43% ± 1.0%).

Fungal infection of the initial grain kernels was approximately 22%. After three months of storage in PICS bags, the infection level remained essentially unchanged in both locations (Fig. 4). In the woven bags stored in Indiana the number of infected kernels increased significantly (P < 0.01) to 32% and to 49% in the Arkansas grain (Fig. 4). The predominant genera identified were *Fusarium*, followed by colonies of *Aspergillus* and *Penicillium*.

### 3.4 Seed germination

The initial germination rate of the maize before storage was 43%. Regardless of bag type or location, germination decreased significantly (P < 0.01) from the initial germination rate after three months of storage (Fig. 4). At the Indiana location, the germination was reduced by 3% in the PICS bags and 12% in the woven bags (Fig. 4), and in Arkansas there was a 12% reduction in the PICS bags and 26% in the woven bags. There was no significant difference (P < 0.01) between the layers within each bag, except in one replicate of the woven bags stored in Arkansas. In this bag, the germination was significantly lower in the bottom layer of the bag. There was no significant difference (P < 0.01) between replicates of each bag type for a given environment.

### 3.5 Aflatoxin

The initial aflatoxin B1 (AFB1) concentration in the *A. flavus*-colonized maize placed in satchels was 13.9 μg of AFB1 per kg of maize (SE = ± 0.65 μg/kg). After three months of storage in Indiana, the AFB1 concentration decreased to 2.07 μg of AFB1 per kg of maize (SE = ± 0.09 μg/kg) and 2.72 μg of AFB1 per kg of maize (SE = ± 0.33 μg/kg) in the PICS and woven bags, respectively. A similar reduction was measured in the Arkansas grain, with 2.28 μg of AFB1 per kg of maize (SE = ± 0.16 μg/kg) in the PICS bags and 2.05 μg of AFB1 per kg of maize (SE = ± 0.12 μg/kg) in the woven bags. For both bag types and both locations, no aflatoxin was detected in the non-inoculated grain placed in satchels adjacent to the *A. flavus*-colonized maize satchels.

## 4 Discussion

Maize grown in the Sub Sahara is often contaminated with aflatoxins due to an ear rot disease caused by *A. flavus* and poor postharvest handling (Atehnkeng et al., 2008, Mukanga et al., 2010, Setamou et al., 1997, Wagacha and Muthomi, 2008). Non-uniformity of the contamination within a grain mass leads to high variability in aflatoxin assessments and results that often conflict with expected norms (Mallmann et al., 2014). The maize used in this study was ideal for addressing the efficacy of hermetic storage. The amount of grain dust and BCFM, which contributed to a U.S. No. 5 grading, increased the vulnerability of the grain to insect and fungal invasion as well as moisture absorption. Additionally, the two storage locations (Indiana and Arkansas) provided environmental conditions that were starkly different. The placement of satchels of aflatoxin-free grain in close proximity to highly contaminated grain allowed us to address the central question of whether aflatoxin accumulation occurs in either the PICS or woven bags during storage.

Placement of temperature/humidity data loggers inside the storage bags provided new information about the changes occurring in response to daily environmental conditions. The diffusivity of heat through bulk maize is influenced by the moisture content and porosity (Kustermann et al., 1981). The diurnal change in the environmental temperature resulted in cyclic heating and cooling of the grain; however, the center of the grain mass, where the data logger was located, never reached equilibrium with the outside temperature. These temperature characteristics where not impacted by the type of storage bag or location. The hermetic seal of the PICS bags shielded the grain from changes in the external relative humidity, resulting in little impact on the RH within the grain mass or in grain moisture content. Within the woven bags, the data loggers did not detect daily changes in external RH; however, the RH gradually increased with time as did the grain moisture content. At the Arkansas location, during a period in September when the external RH was low resulted in a decline in the internal RH. Stored grain can exchange moisture with the environment and reach an equilibrium point (Pixton and Warburton, 1971). Williams et al. (2014) observed that low (29%) RH resulted in the drying of grain stored in woven bags but not PICS bags. We speculate that the September period of lower RH in Arkansas contributed to the lower final moisture content of the grain compared to grain in the Indiana experiment.

Regardless of the higher humidity at the Indiana site and the higher temperatures in Arkansas, aflatoxin was not detected in the grain after three-months of storage in either the PICS or woven bags. Sauer and Burroughs (1980) found that spread of *A. flavus* and aflatoxin accumulation was minimal at 16.5% maize moisture content, but growth and mycotoxin accumulation was extensive at 17.4%. A greater amount of rewetting occurred in the grain stored in the Indiana woven bags (2.9%), which resulted in a final moisture content (15.9%) that was below these minima for *A. flavus* and aflatoxin. We also observed a decrease of 75–80% in AFB1 in the satchels containing the *A. flavus*-contaminated maize. The reason for the decrease is unclear. The only example we found in the literature where aflatoxin contamination decreased was in field experiments conducted in North Carolina (Payne et al., 1988). The authors observed that aflatoxin contamination levels increased in *A. flavus*-infected kernels until maturity and then decreased during the dry-down period.

Although conditions within the woven bags were not conducive for spread of *A. flavus* to non-infected grain, there was a 50% and 130% increase in kernels infected by other fungi in Indiana and Arkansas, respectively. These fungi included *Aspergillus*, *Fusarium* and *Penicillium* species, which have been identified previously in stored maize by others (Adisa, 1994). Many of these fungi are xerophiles capable of growth at moisture levels between 13 and 14.5% (Christensen, 1957).

The optimal temperature for the growth and development of storage insects is between 25 and 33 °C, and growth is slower when temperatures are outside this range (Fields, 1992). Over the three-month storage period, weather at the two experimental sites (Indiana and Arkansas) experienced starkly different temperatures, which resulted in differences in the final insect counts. In Indiana, a temperature above 27 °C was recorded on only one day and nighttime lows below 20 °C were recorded on 51 days. In contrast, Arkansas experienced only 13 nights with temperatures below 20 °C and 66 days with daytime highs above 27 °C. The highest temperature recorded in Arkansas was 34 °C, which was reached on seven days. As a result of these conditions, insects in the Arkansas bags were 34-times (woven bags) and 2-times (PICS bags) higher than in Indiana. The vast majority of the insects were weevil species. Throne (1994) found that maize weevil (*Sitophilus zeamais*) development takes about five weeks, suggesting that under optimum conditions about two to three generations of weevils could develop during the 12-week storage experiment. Throne (1994) also described the propensity of females to lay more eggs under optimal temperature and humidity, which was near ideal in Arkansas and reflected in the large number of insects in the woven bags. Insects found in the PICS bags after the storage period indicate that the initial grain contained weevil and beetle eggs. In Arkansas, where environmental conditions were optimal for development of these eggs into larvae and adult insects, the PICS bags reduced insect development by greater than 80-fold compared to development in the woven bags. Even in Indiana, the PICS bags controlled insect numbers more effectively than the woven bags. These results are in agreement with many studies that have documented the efficacy of PICS and other hermetic bags (Amadou et al., 2016, Baoua et al., 2014, Murdock et al., 2012, Mutungi et al., 2014, Njoroge et al., 2014).

It is a well-documented phenomenon that the respiration of established fungal pockets combined with the respiration of insects can create hot spots within stored grain (Sinha and Wallace, 1966, Wallace and Sinha, 1962). This process of heating was observed in the woven bags stored in Arkansas. Wallace and Sinha (1962) showed that the development of these hot spots leads to the establishment and spread of *Penicillium* and *Aspergillus species* while the viability of persisting field fungi diminishes. Sone (2001) found *A. glaucus* infected 52.0% of kernels after 80 days of storage with weevils in maize at 13% moisture content, 26.6 °C, and 60% RH. Sinha (1984) suggested that the microclimate created by insects increases temperature and relative humidity making conditions conducive to the growth of storage fungi. Sinha and Sinha (1992) found that, with maize stored in ventilated glass bottles without insects, moisture content increased from an initial 13.9%–14.3%. When the maize was infested with *S. oryzae* and *Tribolium castaneum,* the moisture increased to 25%. Furthermore, these researchers reported that, in grain containing insects and inoculated with *A. flavus,* the moisture content rose to 26.4% after 10 weeks of storage. The spread of *A. flavus* to kernels also increased from 8% to 100%. In our study, the woven bags stored at the Arkansas location contained significantly more insects in the bottom compared to the top and middle. Considering that the fungal counts in the bottom layer were 10-fold higher than the top and middle, the combination of fungal and insect activity did not result in grain moisture content in the bottom layer greater than 15.9%, which is below the optimum for *A. flavus*.

In summary, our results show that the use of hermetic storage mitigates many of the environmental effects that lead to the spoilage of grain. Hermetic storage provides a barrier to the exchange with environmental moisture, preventing rewetting that can contribute to the proliferation of storage fungi. Our results also support previous research showing that hermetic storage bags protect maize from insect infestation and development. Although conditions in the woven bags never became conducive for the spread of *A. flavus*, other storage fungi were able to proliferate throughout the woven bags due to optimal conditions of moisture. The placement of *A. flavus*-colonized and non-colonized maize in small satchel provided evidence that *A. flavus* spread and aflatoxin accumulation does not occur if the grain moisture content is not optimal. These results also suggest that protocols for sampling storage must account for high aflatoxin-containing kernels within the bags that can lead to variability and potential erroneous conclusions about the efficacy of hermetic bags. Ultimately, this study demonstrated the use of PICS bags as a practical option for storing and protecting grain from a variety of detrimental environmental effects.
